# Automated home cage monitoring of an aging colony of mice—Implications for welfare monitoring and experimentation

**DOI:** 10.3389/fnins.2024.1489308

**Published:** 2024-10-29

**Authors:** Joanna L. Moore, James Kennedy, Abdul-Azim Hassan

**Affiliations:** ^1^Biological Services, University of Glasgow, Glasgow, United Kingdom; ^2^Research Statistics, GlaxoSmithKline, Stevenage, United Kingdom; ^3^Respiratory Immunology Biology Unit, GlaxoSmithKline, Stevenage, United Kingdom

**Keywords:** animal welfare, aging, home cage monitoring, rodent behavior, cage changing, circadian rhythm, acclimatization period, activity-biomarkers

## Abstract

**Introduction:**

Our understanding of laboratory animal behavior and the implications of husbandry activities on their wellbeing remains incomplete. This is especially relevant with an aging colony as their activity patterns may change as they mature. Home Cage Monitoring (HCM) provides valuable insights into mouse activity within the animal's own environment and can shed light on acclimatization periods and responses to husbandry activities such as cage changing. The aim of this study was to monitor and explore changes in the activity and rest disturbance (RDI) patterns of an aging colony of male and female C57/BL6 mice.

**Methods:**

The mice were housed in the Digitally Ventilated Cage^®^ system, for up to 18 months of age. Data was then downloaded to investigate how the activity patterns and RDI of the mice changed over time. Habituation, aging and cage change assessments were conducted using linear mixed models, while cage separation and stereotypic behavior investigations were conducted by visual inspection of the data.

**Results:**

As expected during the study, mice were less active during the light phase compared to the dark phase. However, on arrival mice displayed heightened activity and RDI during the light phase and reduced activity and RDI during the dark phase, taking several days to adjust to baseline “acclimatized” patterns. With age, overall activity significantly decreased from 5 months until 14 months of age, after which it increased back toward baseline levels. We also observed activity spikes during our monitoring of this colony. Prolonged housing can lead to alarming stereotypic behaviors in animals. Cages of mice flagged for potential stereotypy displayed sustained activity spikes in the light and dark phases. Spikes in activity during the dark phase were much more pronounced than in the light phase. Cage changing led to an increase in the light phase activity and RDI compared to the previous day, with no observed difference in the dark phase post-cage change. This effect remained consistent as the animals aged.

**Discussion:**

This study explores changes in the activity patterns of an aging colony of male and female C57/BL6 mice housed in the Digitally Ventilated Cage^®^ system. We identified distinct aging phases concerning activity and RDI differences and a potential new welfare application for the DVC^®^, specifically for early detection of stereotypy. In conclusion, the adoption of HCM systems should be considered for long-term animal housing from both a welfare and behavioral perspective.

## Introduction

Increasing the understanding of mouse ethology enables researchers to make more objective interpretations of data. This is especially important in biomedical scientific research because it may offer more robust, translatable data. Typically, the starting point for the majority of investigations is with the background strains to understand and compare the effects of gene manipulations on mouse ethology. These effects include aspects such as fertility (Vasudevan et al., 2010), aggression (DeVries et al., 1997), and disease progression (Manocha et al., 2019) or to use as a study control. The increasing number in ethology driven papers using Home Cage Monitoring (HCM) indicates the demand for more work in this area. One reason for this trend may be the focus on background strains such as the widely used C57/BL6 mice to offer a more standard activity profile. This data could be used to form baseline “normal expected activity” for the Genetically Modified (GM) strains which have been derived from these background strains. Establishing such baselines is crucial for two main reasons: (1) it increases our insight into how far a GM strain deviates from normal behavior compared to its background lineage, and (2) it provides further understanding of typical laboratory mouse behavior.

Laboratory mice are put through a wide range of different housing, husbandry, and experimental procedures throughout their lifetime. Many of these can cause them a level of anxiety, which is difficult to objectively measure. The use of HCM has enabled us to learn more about the response of mice to routine housing, husbandry, and experimental procedures. The value of this knowledge increases as scientific evidence grows, indicating that standard factors used in maintaining their wellbeing can have detrimental effects even before experiments commence. For example, non-aversive handling can decrease anxiety-like behaviors such as reluctance to interact with handlers (Sensini et al., 2020) and the observation of a significant increase in activity during the light phase, when mice are usually resting, in response to routine cage changing (Pernold et al., 2019).

Another growing area of focus is the inclusion of both male and female mice in *in vivo* studies. This inclusion can increase translatability (Karp and Reavey, 2019) and reduce the wastage of animals, making it more important to understand the fundamental similarities and differences between the responses of male and female mice to interventions.

Despite the increase in HCM investigation a recent meta-analysis has revealed various gaps in the assessments of home cage monitoring. In the past 10 years both sexes were used in only 28% of the 521 studies that were analyzed. Animal housing densities in those studies were mostly performed for singly housed animals and were therefore unlike the optimum situation for housing of rodents. Monitoring longer than 3 months was rare and abnormal behaviors were not often paired with HCM data (Kahnau et al., 2023).

Assessing how mice react to a standard laboratory housing regime can be challenging, as mice are generally only in the laboratory for specific experimental purposes. It is even less common for mice to be housed in a consistent state for studies spanning over an 18-month period. As part of a larger study to biochemically characterize a genetically altered animal model with a knock-in present in neurological disorders of humans (write-up in progress), the wild type (WT) mice (generically C57/BLN) were considered ideal candidates for longitudinal behavioral analyses. These animals were only subjected to cage changing and routine health monitoring, which included non-aversive handling using a tunnel for cage changing, body weight, and health observations. Keeping these animals in a familiar environment (home cage monitoring) for this extended period revealed patterns that would otherwise be unmeasurable.

In this report of our observational study, we directly address the gaps highlighted by the meta-analysis of Kahnau and colleagues. We present the results of activity monitoring of male and female mice housed long-term in a standard Individually Ventilated Cage (IVC) on a Digitally Ventilated Cage rack (DVC^®^). The procedures we have investigated are routine for most laboratory mice including changes in activity over the acclimatization period and their response to cage changing. We include investigations of Regularity Disruption Index (RDI) data generated from the DVC^®^, and the general activity profile of the mice across an 18-month period to determine if significant differences in their response to long term housing can be observed. The generation of this type of data not only enhances animal welfare and aids in the establishment of humane endpoints, but it also produces a large amount of information regarding rodent life in the home cage.

## Methods

### Animals

All animal studies were ethically reviewed and carried out in accordance with Animals (Scientific Procedures) Act 1986 and the GSK Policy on the Care, Welfare, and Treatment of Animals.

The data in this study was derived from a control group of animals from a larger study, which adhered to all relevant ethical standards. Because the DVC^®^ system stores all; activity data it allowed us to optimize data collection without compromising animal welfare or using more animals. The activity data we analyzed was only available because the mice were monitored using this system.

Mice were transferred to the GSK facility (Stevenage, UK) from The Jackson Laboratory (Bar Harbor, USA), on arrival, the mice were health checked carefully and group housed in XT GM500 Digitally Ventilated Cages (DVC) (Tecniplast S.p.A, Italy). DVCs are made from a clear Polypropylene plastic base with a stainless-steel bar lid, and internal measurements of 35.5 × 17.5 × 12 cm. Environmental enrichment was provided in all cages with Lignocel BK8/15 bedding (IPS, UK), a mouse igloo, an aspen wood chew block (Datesand, UK), half a Lignocel Large Wood Wool disc (IPS, UK), a mouse mini fun tunnels (LBS, UK) and a mouse handling tunnel.

The health status of the mice complied with “*Federation of European Laboratory Animal Science Associations Health Monitoring recommendations for mice”* (Mähler Convenor et al., 2015). The cages were randomized to their position on the rack using a simple randomized list generated using internal software. Rack positions were numbered and randomized to provide the locations of the cages as they arrived into the lab. All mice were individually identified using ear marks. The light cycle was on a 12:12 light: dark phase, with light phase from 6 a.m. to 6 p.m. and a gradual increase or decrease of lighting over a 10-min dawn; dusk period. The room temperature was 21–24°C, humidity was 55 ± 10%, and there were 75 air change per hour in the DVCs^®^. The DVC^®^ is an automated animal activity recording system that records data every 0.25 ms using an externally place capacitance board made up of twelve electrodes. The sensors are activated and deactivated by the mice as they locomote around the cage using a non-intrusive method (Iannello, 2019). RDI is a metric that measures irregularities of a time series (home cage activity in this paper) and is not influenced by the absolute amount of the activity itself. When minutes of activity are similar to each other, then RDI tends to be smaller and vice versa.

At the start of the study all males and female mice were housed in trios. The mice arrived over two different dates, Cohort 1 arrived in July 2021 and Cohort 2 arrived exactly 4 weeks later. During the study, where fighting bouts leading to injury occurred (male mice only), mice were separated. Mice were fed *ad libitum* with 5LF2 irradiated diet (IPS) via food hoppers at the rear of the cage, and *ad libitum* animal grade filtered reverse osmosis and UV treated drinking water via bottles attached to the front cages, food and water was monitored by the staff and the DVC^®^ system.

GSK is committed to the replacement, reduction and refinement of animal studies (3Rs). Non-animal models and alternative technologies are part of our strategy and employed where possible. When animals are required, application of robust study design principles and peer review minimizes animal use, reduces harm and improves benefit in studies.

### Statistical methods

All data formatting and analyses were conducted in R version 4.1.1 (2021-08-10) and RStudio 2021.9.0.351.

Mice were kept in their stable groups for the duration of the study unless they required separation due to fighting. On the day of arrival mice were brought into the room in the late morning, therefore the light phase data on Day 1 was shorter.

#### Habituation assessments

Cage activity and RDI were aggregated on an hour-by-hour basis, defined from the first minute to the last minute of each hour (e.g., 11:00–11:59). Linear mixed models were used to investigate the length of time it took the mice to display consistent, cyclic patterns of behavior. These models used hour, sex, and the day after housing as fixed effects, with the hour including linear, quadratic, and cubic terms. The models included a two-way interaction term between the hour and the day of housing. Separate models were created for each of the first 5 days of housing including data from the day 31 of housing (~1 month later) for reference. Analysis of variance was used to calculate whether the inclusion of the two-way interaction explained a significant proportion of the variation within the data. This had the effect of quantifying if there was a significant change in activity and RDI between one of the first 5 days of housing and day 31 of housing.

#### Aging assessments

Cage activity and RDI were aggregated over each month of age from 2 and 18 months, on an hour-by-hour basis, defined from the first minute to last minute of each hour (e.g., 11:00–11:59). Linear mixed models were used to estimate and compare movement behavior between months of age, mouse sex (male and female) and cage density (the number of mice within a cage). All *p*-values from these comparisons were multiplicity adjusted using the Benjamini Hochberg method. The models used sex, hour, and cage density as fixed effects, with hour including linear, quadratic, and cubic terms. The models included three-way interaction terms between age, sex, and hour and between age, hour, and cage density. Cage ID was included as a random effect to account for correlation between repeated measures from the same cage. Note that female cages contained two or three mice whereas male cages contained one, two or three mice (as a result of cage separations due to fighting). Data from days when there were bedding changes or cage separations were excluded from the analysis (where applicable).

#### Cage separations investigation

Cage activity and RDI were aggregated on an hour-by-hour basis, defined from the first minute to the last minute of each hour (e.g., 11:00–11:59). Data from cages where cage separations occurred (due to fighting) were visually inspected to understand if there were any noticeably irregular patterns of behavior leading up to the cage separation. This was done by visually comparing against data from cages where separations did not occur (within the same period of time). Fighting occurred in the male cages only.

#### Stereotypic behavior investigations

Cage activity and RDI were aggregated over each day by calculating the average activity/RDI during the light and dark phase periods, defined from 6:00 to 17:59 and 18:00 to 5:59 (of the following day), respectively. Data from cages where stereotypic behavior was suspected was visually inspected and compared against cages where stereotypic behavior was not suspected. This applied to two female cages, with stereotypic behavior not having been suspected in any other cages.

#### Post cage change behavioral assessments

Cage activity and RDI were aggregated over each day by calculating the average activity/RDI during the light and dark phases, defined from 6:00 to 17:59 and 18:00 to 5:59 a.m. Linear mixed models were used to investigate the effect of bedding changes on movement behavior on and after the day of a bedding change, with all *p*-values being multiplicity adjusted using the Benjamini Hochberg method. The models used day, sex, age (in months), and cage density (the number of mice in a cage) as fixed effects. The models included a three-way interaction term between day, sex and age and a two-way interaction term between age and cage density. Cage ID was included as a random effect to account for correlation between repeated measures from the same cage. Data from days where cage separations occurred were excluded from the analysis (where applicable).

## Results

### Mice exhibited differential cage activity and RDI profiles upon arrival into the unit

**Figure 1:**
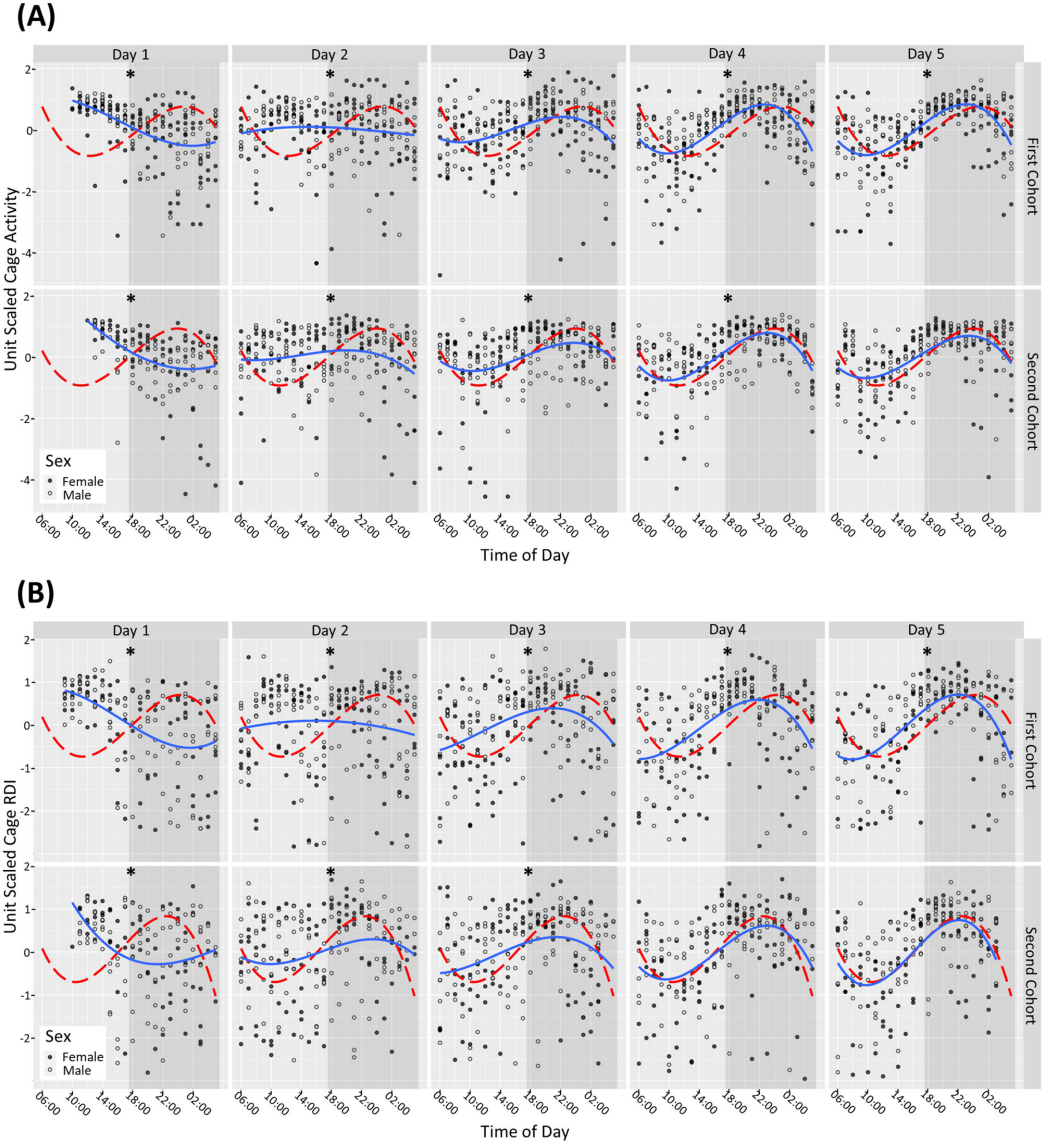
Mice require 4+ days after arrival into the facility to reach normal rhythms of activity and RDI. Cage activity and RDI were measured using scaled activity **(A)**, scaled cage RDI **(B)** across the day of arrival with data aggregated in hour buckets. Light gray areas depict the light phase, and the shaded areas depict the dark phase. A summary of the cage activity and RDI from the first 5 days of housing. The first day of housing is indexed as Day 1. The estimated activity/RDI profile for the day of interest (blue line) is compared with the activity/RDI profile across the 24-h period at day 31 (red dashed line) for male (o) and female (•) mice combined. The top graphs represent Cohort One, bottom graphs represent Cohort Two which arrived into the facility at ~1 month later. Days with significant differences to reference line (Day 31) are marked as *P < 0.05.

On day of arrival (D1) male and female mice had increased activity and RDI during the light phase and reduced activity during the dark phase compared to the subsequent light and dark phases (per hour tested) on day 31. Dark and light phase activity and RDI became more consistent from D3 and continued to normalize on subsequent days (lower during the light phase compared to the dark phase irrespective of sex). For the second cohort, by day 5 there was no statistically significant difference observed between the male and female mice in their RDI and activity profile compared to D31. For the first cohort, even at D5 there were still significant differences observed with the male and female mice in their activity profile (Figure 1A) and RDI compared to D31 (Figure 1B).

### Over an 18-month period, cage activity and RDI profiles differed between age, number of mice per cage and sex

**Figure 2:**
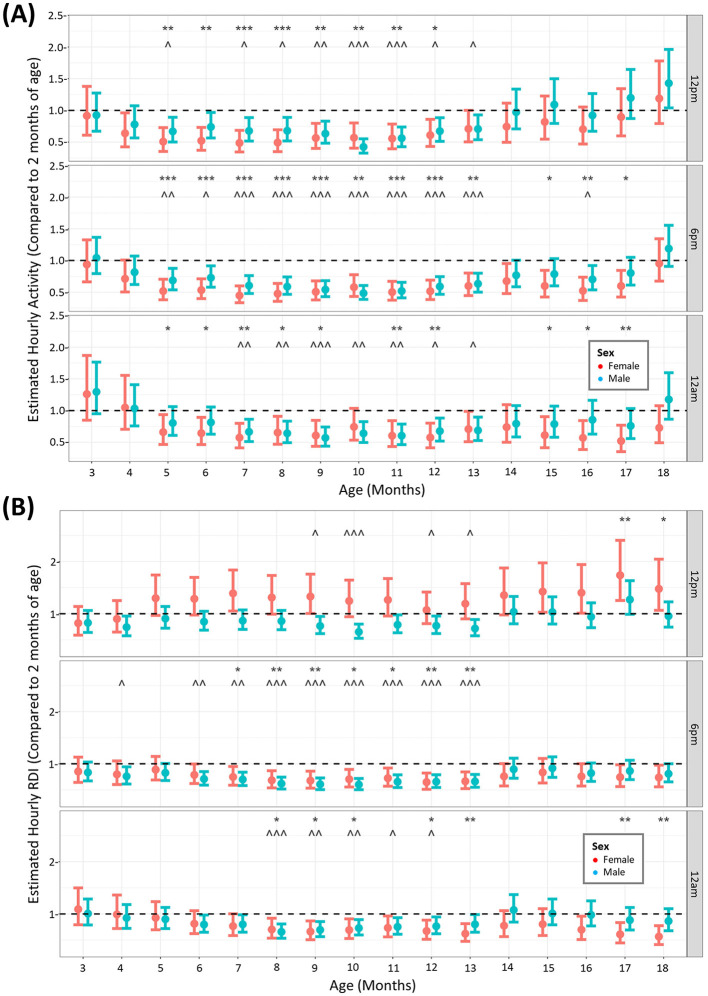
Increased age results in reduced activity and RDI of mice until 14 months where it returns back toward baseline levels. The estimated cage activity **(A)** and RDI **(B)** at 12 p.m., 6 p.m., and 12 a.m. in female (•) and male mice (•) for 18 months. Data are presented as the estimated mean ± 95% confidence interval. Months with significant differences to baseline for female mice are marked as *P < 0.05, **P < 0.01, ***P < 0.001. Months with significant differences to baseline for male mice are marked as ^∧^*P* < 0.05, ^∧∧^*P* < 0.01, ^∧∧∧^*P* < 0.001.

Animals were monitored using the DVC^®^ over the study period of 18 months to elucidate whether aged animals have different profiles of activity and RDI. Although the data was modeled continuously, we compared the activity at three key timepoints: 12 p.m. (11–11.49) from our observations of the daily rhythm (Figure 1) was the time when animals were least active (5–5.59 a.m.) 6 p.m. is the beginning of the dark phase and 11–11.59 a.m. (12 am) was when animals were most active. We compared the estimated data at all months in the study against the estimated activity or RDI at 2 months of age, the month at which the animals arrived into the facility. When looking at activity (Figure 2A) at these timepoints, although there are fluctuations in some months between male and female, overall activity significantly decreases from 5 months of age until 14 months of age (inclusive). After this it starts to increase back toward baseline levels. For activity this is true for all timepoints. RDI (Figure 2B) is more time dependent and is consistently significantly different from baseline between 9 and 13 months (inclusive) of age for at least one sex of mice. The same trend of increased elderly activity is observed on the whole for RDI also, where the animals at their oldest tend to have similar RDI as that at 2 months of age.

**Figure 3:**
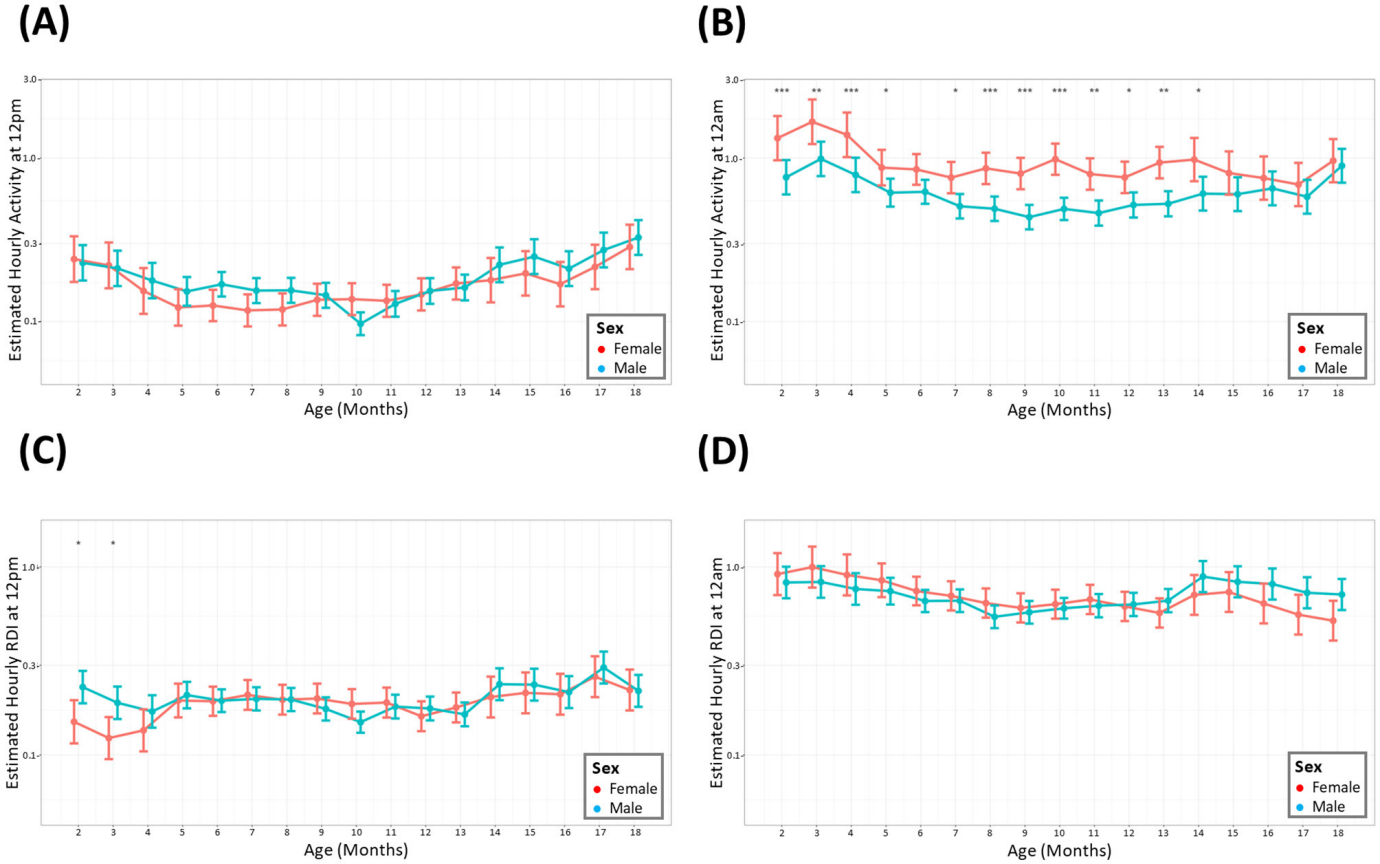
Female mice have consistently higher levels of nighttime activity. The estimated cage activity and RDI at midday **(A, C)** and midnight **(B, D)** in female (•) and male mice (•) for 18 months. Data are presented as the estimated mean ± 95% confidence interval. Months with significant differences between male and female are marked as *P < 0.05, **P < 0.01, ***P < 0.001.

The average hour-by-hour, monthly activity across the entire study period indicated that males showed similar activity to females in the light phase, depicted by the activity at its lowest point (12 p.m.), (Figure 3A). Females were consistently more active compared to males during the midnight timepoint. The statistical significance of this difference was maintained until 15 months after which there was no statistical significance to the elevated female activity (Figure 3B). RDI was similar between the sexes throughout the study apart from the initial months of the study where females had a small but significant lower RDI at 12 p.m. (Figures 3C, D).

**Figure 4:**
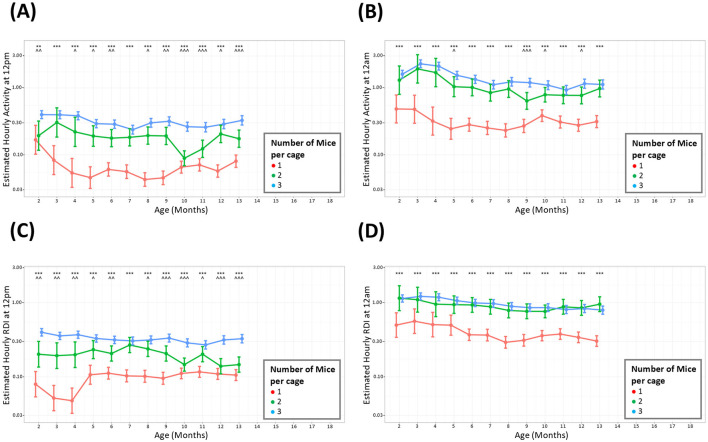
Cages with more mice have consistently higher levels of activity and RDI. The estimated cage activity and RDI at midday **(A, C)** and midnight **(B, D)** in cages with; three mice (•), two mice (•), and one mouse (•) for 13 months. Data are presented as the estimated mean ± 95% confidence interval. Months with significant differences between three mice per cage and one mouse per cage are marked as *P < 0.05, **P < 0.01, ***P < 0.001. Months with significant differences between three and two mice per cage are marked as ^∧^*P* < 0.05, ^∧∧^*P* < 0.01, ^∧∧∧^*P* < 0.001.

Mice were housed in groups of three at the start of the study but occupancy per cage was reduced when welfare concerns necessitated removal of a mouse or splitting of cages. Mice were only singly housed when necessary to prevent further fighting and were used as soon as practical and were taken off study as soon as possible. The analysis was only conducted up to 13 months due to the small number of cages of singly housed mice present on study after this timepoint. The average hour-by-hour, monthly activity across the whole study period indicated that during the light and dark phase, cages with fewer animals showed lower overall activity than those with three mice per cage (Figures 4A, B). A significant difference in cage activity was seen at all months when comparing cages with three mice against cages with 1 mouse. Cages with two mice also displayed consistently lower overall activity compared to cages with three animals, however this was statistically different fewer times particularly at night. Investigations of RDI when comparing cages with different number of occupants also show these patterns where cages with singly housed mice showed less irregularity (lower RDI) than cages with multiple occupants (Figures 4C, D).

### The DVC^®^ system may be used to investigate abnormal behavior and welfare concerns

**Figure 5:**
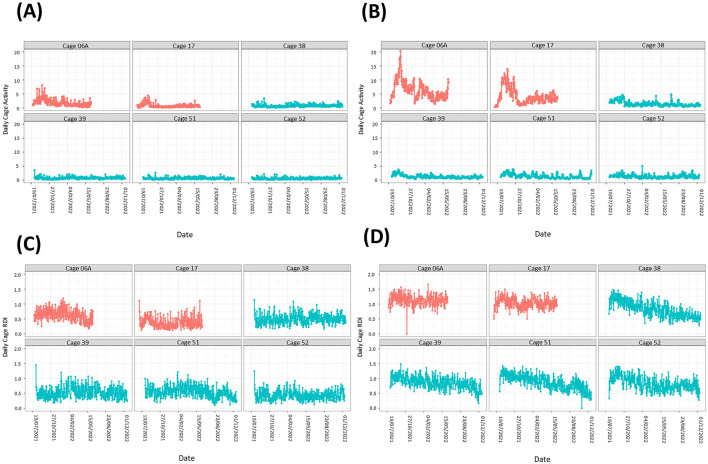
Cages containing animals exhibiting stereotypic behavior show large spikes in activity but no obvious differences in RDI. Activity **(A, B)** and RDI **(C, D)** profiles of cages with mice showing stereotypic behavior (red) alongside profiles of cages with no stereotypy observed (blue). Light phase measurements **(A, C)** as well as dark phase measurements **(B, D)** are presented.

On two occasions we observed stereotypic activity in two separate cages containing female mice. Analysis of the data from those cages showed sustained activity spikes in the light and dark phases compared to cages with no stereotypic activity reported (Figures 5A, B). Spikes in activity during the dark phase (Figure 5B) were much more pronounced than in the light phase (Figure 5A). In contrast, RDI data for both cages were similar to cages where no stereotypic behavior was observed (Figures 5C, D).

**Figure 6:**
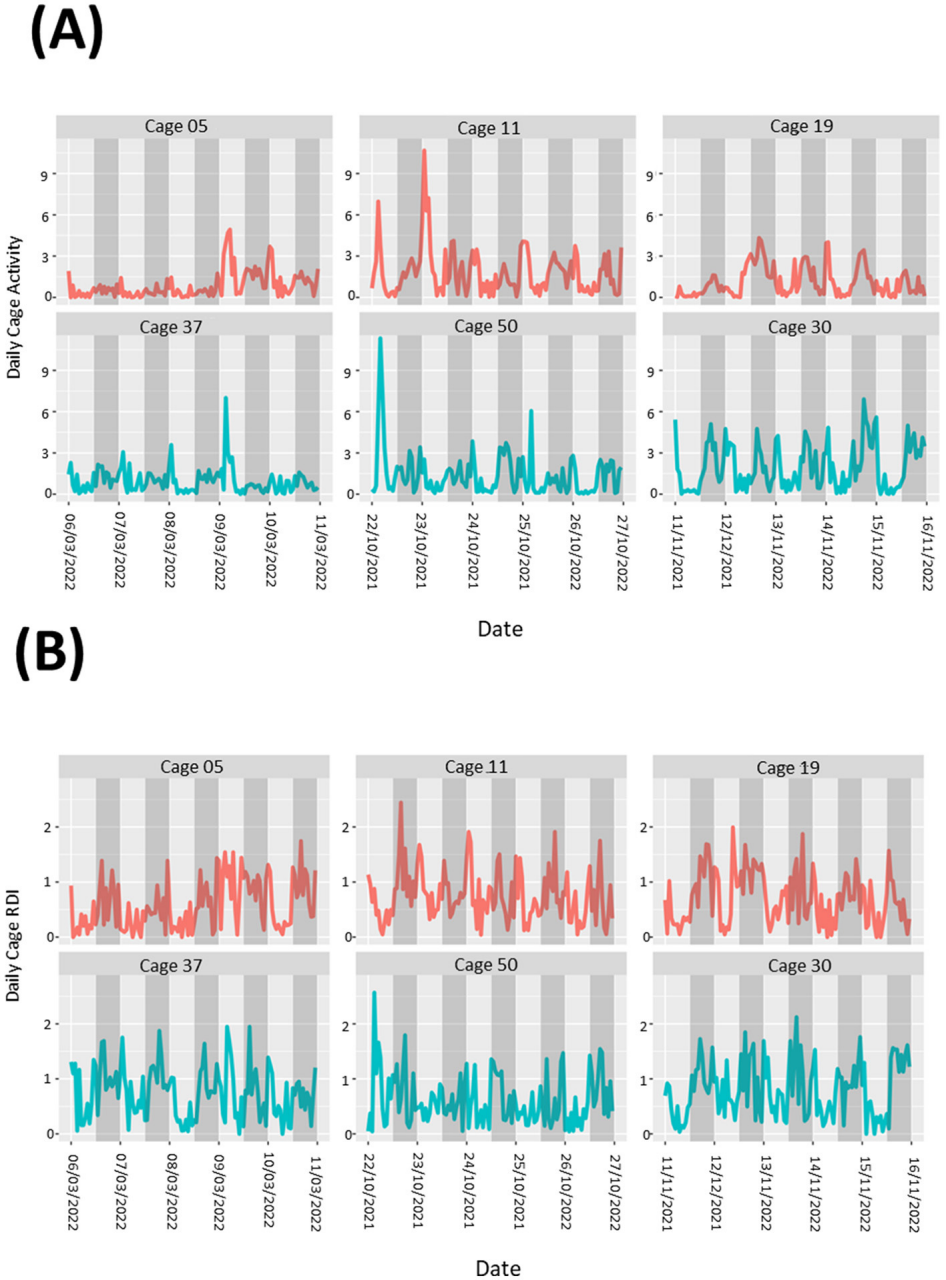
Cages containing animals exhibiting fighting behavior show no obvious patterns to differences in overall cage activity or RDI. Activity **(A)** and RDI **(B)** profiles of cages with mice that required separation due to fighting (red) alongside profiles of cages with no instances of fighting (blue). Measurements of 5 days leading up to separations are presented.

During the study there were occasions when male mice were removed from their group housing and singly housed. Data was analyzed in the 5-day period leading up to a cage separation, however we were unable to determine any noticeable pattern to the differences in the activity (Figure 6A) or RDI (Figure 6B) profiles compared to similar cages where fighting and separation did not occur.

### Changing the cage of mice elicited increases in light phase activity and RDI profiles

**Figure 7:**
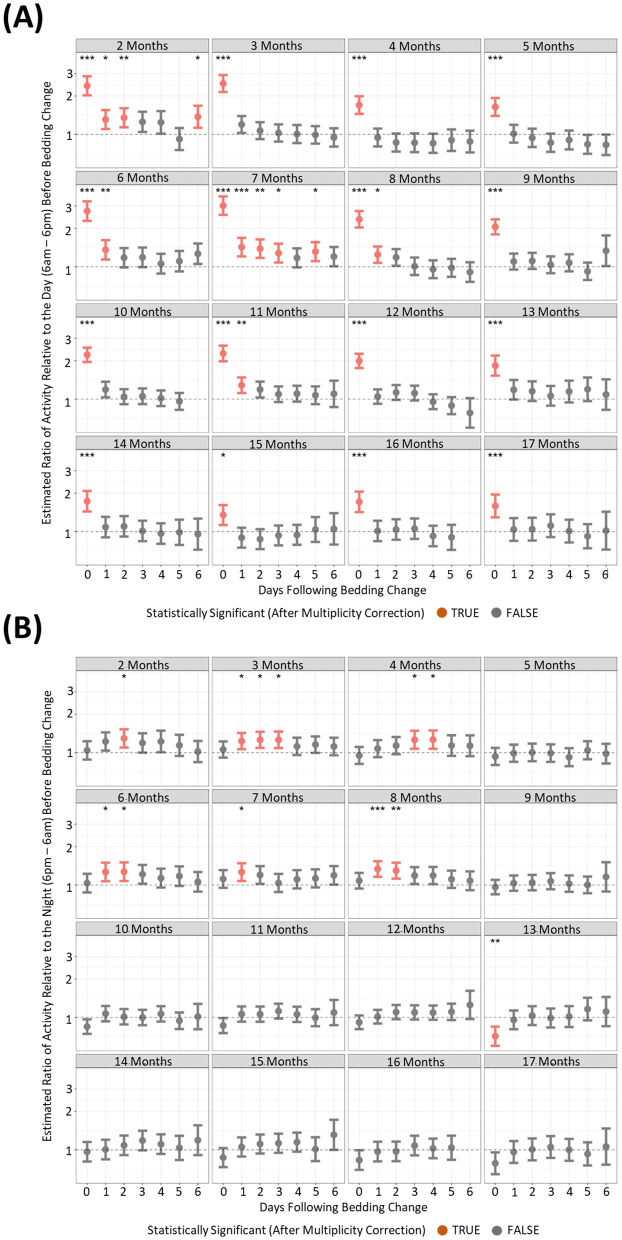
Higher levels of daytime cage activity are observed after a cage change when compared to cage activity prior to a cage change. The estimated ratio of cage activity in the “daytime” **(A)** and “night” **(B)** in cages containing mice up to 17 months of age. Data are presented as the estimated mean ± 95% confidence interval. Timepoints with significant differences compared to the day prior to cage change are marked in red and as *P < 0.05, **P < 0.01, ***P < 0.001.

**Figure 8:**
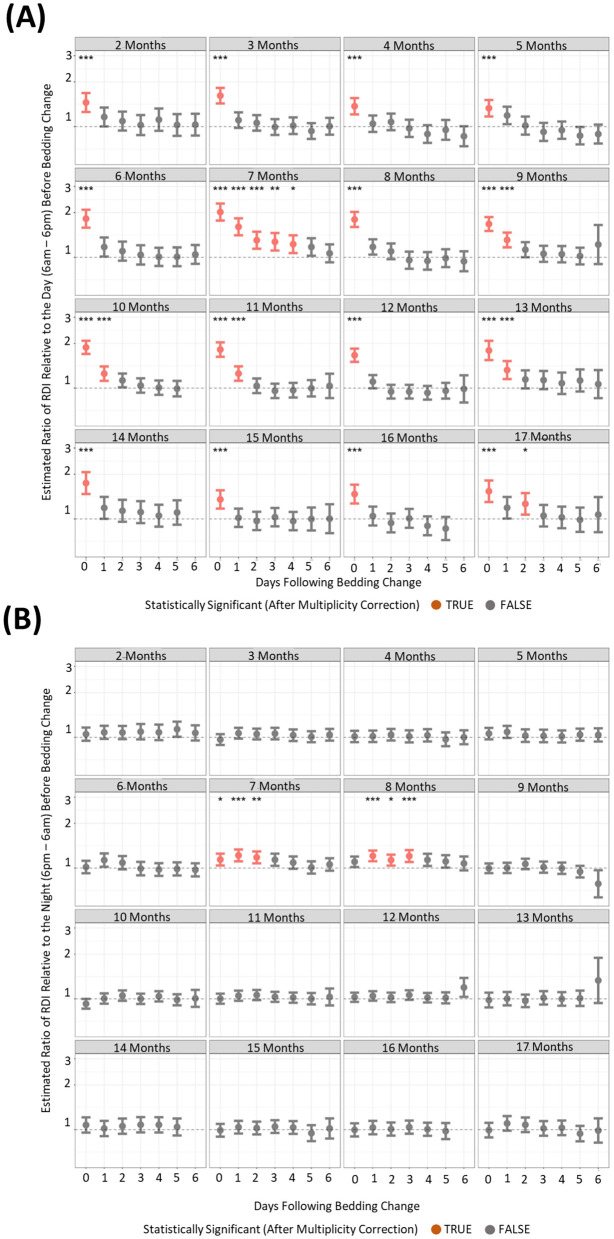
Higher levels of daytime cage RDI are observed after a cage change when compared to cage RDI prior to a cage change. The estimated ratio of cage RDI in the “daytime” **(A)** and “night” **(B)** in cages containing mice up to 17 months of age. Data are presented as the estimated mean ± 95% confidence interval. Timepoints with significant differences compared to the day prior to cage change are marked in red and as *P < 0.05, **P < 0.01, ***P < 0.001.

To understand if behavioral changes are apparent after the changing of housing, we compared activity and RDI of cages on the six subsequent days after a cage change compared to the day prior. There were many differences between the months where increased activity was significantly observed post cage change (Figures 7A, B). However, there was only one timepoint that consistently showed increased cage activity and that was on the day of the cage change (D0) where activity was 2–3-fold higher than the activity of the day prior to cage change (Figure 7A). Dark phase activity was not significantly different in 9 of the 15 months measured at any of the timepoints (Figure 7B). Similarly light phase RDI was 1.5–2-fold higher consistently on the day of cage change (Figure 8A) but not consistently on any other day nor in the dark phase (Figure 8B).

## Discussion

This paper examines patterns of activity in wild type mice aged for 18 months assessing changes during acclimatization, aging, potential humane endpoints, and cage changing, during this extended period. Typically, laboratory mice are housed for up to 6 months, making this aging study a unique opportunity to utilize the Digital Ventilation Cage (DVC^®^) as a Home Cage Monitoring (HCM) tool.

In addressing the ethical implications of long-term housing and monitoring (Baumans, 2007; Bellantuono et al., 2020), it is crucial to recognize that mice held into old age require extra care and increased monitoring due to age-related health concerns. Some of these health issues include obesity, reduced activity stemming from poor bone density, decreased eyesight, and diminished self-grooming abilities. Additionally, aged mice may experience weight loss and develop lumps or other health complications. Therefore, our study prioritizes the welfare of these animals, ensuring that appropriate measures are taken to monitor and care for them throughout their lifespan. The age of animals in experiments is an important factor, as a young mouse (fewer than 6 weeks old) may not correlate directly with an adult human, and there is no clear consensus on how mouse age translates to human age (Jackson et al., 2017). By investigating the aging process, researchers could begin to make more precise assumptions regarding the generalization of age.

The life of a mouse in a cage adheres to a clear diurnal rhythm of activity changes, with 12 h of light and 12 h of darkness. The peak of activity is observed in the dark phase and the lowest activity is seen in the light phase (Pernold et al., 2019). The regularity of activity here measured by RDI, followed a similar pattern (Golini et al., 2020). RDI was a good indicator of rest periods, particularly in the light phase as the metric measures irregularities of a time series. When minutes of activity are similar to each other, then RDI tends to be smaller and vice versa.

On D1, when the mice were brought into the facility, they exhibited elevated activity during the light phase and reduced activity in the dark phase, which can be attributable to travel (Obernier and Baldwin, 2006). They also displayed elevated daytime RDI and low dark phase RDI indicating a lack of regularity at traditional rest times with more consistent rest activity during traditional active periods, again likely due to their recent arrival. From Day 4 onwards, both male and female mice demonstrated more consistent activity and RDI patterns. However, when looking at the profiles of the two separate cohorts of mice, the first cohort remained statistically different in terms of activity and RDI compared to D31 (used as a reference), for the first 5 days assessed. The second cohort seemed to acclimatize better as from D4 the RDI and activity was no longer statistically significantly different to D31. D31 was used as a reference because it was assumed that by 1 month in the facility that the activity patterns would have become regular. At day 31 the data indicated a clear anticipation with a gradual increase in activity at the 2 h before the lights-off phase (red line Figure 1A). A peak in activity 2–4 h into the dark phase was observed followed by decreased activity in anticipation of the light phase.

This increase in activity, as a response to the travel, is one of the reasons mice are usually given a 5-day habituation period in their new environment. Other publications that have explored physiological changes in mice post-transportation have found that in a group of Balb/C male and female mice these levels stabilized within a few days (Bundgaard et al., 2012). In a study utilizing HCM and other physiological measurements, one group found that activity levels such as rearing, grooming increased even when mice were transported between rooms, these activities stabilized within 3 days and serum corticosterone stabilized after 24 h (Tuli et al., 1995). The data from our study confirmed that this time frame is not always sufficient for mouse activity and RDI to stabilize, but it can vary between cohorts. Acclimatization after extensive travel by air and road, as was the case with these mice (USA to the UK) may require a longer period of time which should be further investigated.

When assessing animals over the 18-month period, comparing to the activity in the animal's first month of arrival, activity decreased from around 5 months of age until 13 months of age, at all timepoints quantified. This was observed in both males and females. Interestingly, activity returned to early levels after 14 months until the end of the study, particularly in males, although we must be mindful that at the end of the study more males were individually housed. This was unexpected as one would expect activity levels to continue to decrease with age until a plateau rather than the rebound increased activity seen here at the late stage. Other studies in wildtype mice housed on the DVC^®^ system have only monitored the animals for 24 weeks (~6 months), in which activity declines from 22 weeks (~5 months of age) (Golini et al., 2020). This is in agreement with the early part of our data set. We can therefore potentially separate the life of the mouse into three phases of activity patterns, an early high activity phase, a middle age declining activity phase (5–13 months of age) and an elderly rebound higher activity phase (14–18 months of age), however, more work needs to be completed to further validate this finding. In terms of RDI, the timepoint in the middle of the day had months in the elderly phase that were significantly higher than at 2 months of age. This was observed in females only, indicating impaired daytime rest at advanced age. Sleep disturbances have been recognized as premorbid signs of disease and increased daytime RDI has been observed in the SOD1G93A mouse model for ALS as a digital biomarker prior to neuromuscular deterioration (Golini et al., 2020) as well as in the mouse destabilization of the medial meniscus model of osteoarthritis (Ai et al., 2023). We performed no further assessments in these mice apart from HCM and so were unable to deduce musculoskeletal issues encountered in the females with increased daytime RDI. RDI at the other timepoints (and in males at 12 p.m.) was decreased in a similar fashion to activity with age, with a similar rebound effect at 14 months onwards. Both studies that reported increased daytime activity also conducted classical motor experiments on their animals, including the rotarod and grip strength tests (Golini et al., 2020; Ai et al., 2023). Applying similar experimental protocols to our aged animals exhibiting daytime activity and RDI disturbances may uncover these DVC endpoints as early markers for musculoskeletal or neuromuscular dysfunction in rodents.

It is important to note that the number of cages in our study decreased at later time points due to the periodic removal of animals for other endpoints. While this was partially accounted for by the inclusion of “cage density” and “cage ID” as factors in the models, this may still have been a confounding factor in our analysis between the later and earlier months and therefore a limitation of the study. Although this was an analysis with a “within-subjects” design, the addition a control group, such as non-aging mice, may have also increased our ability to conclusively attribute the observed changes to aging. To validate and enhance the utility of this system for assessing age-related dysfunctions in rodents, future studies should incorporate similar group sizes, more control groups and employ additional traditional experimental assessments.

During the light phase (quantified at 11.30–12.30 p.m.), no discernible difference was observed between the activity levels and RDI of male and female mice throughout the 18 months. During the dark phase, female mice exhibited a higher level of activity compared to male mice. This has been observed in other studies, where female mice had increased locomotion, higher climbing bouts and increased wheel running activity compared to their male age matched counterparts (Reiber et al., 2022; Bains et al., 2023). Studies using the DVC^®^ set up have also shown significantly elevated activity in females compared to males, although their significant increase in the light and dark phases may be due to differences in analysis methods (Pernold et al., 2019).

To understand whether cages with different densities differed in their activity and RDI, the data was analyzed by grouping cages per number of occupants. Mice were housed in groups of three at the start of the study but occupancy per cage was reduced when welfare concerns necessitated removal of a mouse or splitting of cages. Male mice were singly housed to prevent further fighting and were taken off study as soon as practical. The analysis for this part of the study was conducted up to 13 months due to the small number of singly housed mice after this timepoint. Throughout the light and dark phases, an anticipated difference was observed between cages with singly housed animals and cages of group-housed mice. The difference was discerned between pairs and trios also, however there were several instances when trio cages had similar levels of activity and RDI to cages with pairs. It has been well-documented that social deprivation leads to increased anxiety and depressive-like behaviors (Chourbaji et al., 2005; Berry et al., 2012) and one would expect that this may translate to differences in home cage activity. It is worth noting that the measurements taken in our study are measurements per cage rather than per animal. With that in mind, although there are significant differences in activity and RDI with different cage densities, overall, the differences are proportional to the number of animals per cage, i.e., a cage containing one mouse has ~30% the activity of a cage with three mice.

Stereotypic behavior has been well-published and is generally defined as when animals repeat a behavior continuously with no purpose or function (Ellenbroek and Cools, 1993). On two different occasions we observed stereotypic activity in two separate cages containing females. This was first flagged by a technician and confirmed when a video camera was used to record the in-cage activity. During the light phase, the mice exhibited minimal stereotypy. Upon reviewing the data from the DVC^®^, it was noted that during the dark phase, the activity biomarker signal was extremely elevated. Consequently, the mouse was excluded from the study and humanely euthanized. The use of the DVC^®^ technology facilitated faster build-up of information pertinent to the welfare concerns, compared to the conventional visual observation techniques. There are significant impacts on the psychological state of laboratory animals generally (Miczek et al., 2001; Balcombe, 2006; Gross et al., 2012). Stereotypic behavior is manifested in diverse ways, including, but not limited to, circling with no purpose, overgrooming to the extent where skin damage may occur, heighted aggression and bar chewing. It does not manifest in all animals in a cage and can be hard to observe in mice as they are often sleeping during the day and when they are disturbed, they may not immediately display stereotypic behavior. In our study, the two female mice displayed circling stereotypic behaviors. This continuous movement is perhaps the reason for a lack of difference in the RDI of the cages of these animals when compared to cages where stereotypy was not observed. The increased activity was clear and the DVC^®^ data allowed us to establish the length of time that the stereotypic behavior had occurred for. With the ability of HCMs to send out health alarms, threshold activity levels should be considered to allow early detection of abnormal activity and timely intervention.

Male mouse housing can be further complicated due to their heightened aggression toward their conspecifics. Even in carefully managed colonies, increased fighting may occur, which could lead to the separation of group-housed male mice into individual housing (Van Loo et al., 2002; Marashi et al., 2004; Kaliste et al., 2006; Lidster et al., 2019). Throughout our study, some male cages required the occupants to be separated due to instances of conflict. With the length of our study, we were able to monitor this on several occasions over a period of 15 months. When looking back at the data from those cages, the activity profiles and RDI of those enclosures did not exhibit any observable differences to those appearing to live in harmony. This observation could potentially be attributed to the methodology employed in the analysis of the cage data, which included all the sensors analyzed in hour buckets of data. A more precise outcome might have been achieved through a narrower time frame or the use of fewer sensors. A more targeted approach, however, would require the use of cameras to narrow the timespan and enable the identification of specific sensors activated during fights. The brief episodes of chasing and conflict were not as prolonged or intense as the stereotypic behavior we observed. It is also likely that a sample size of five is simply not large enough for such an investigation to reach sufficient conclusions and enable future predictions.

One of the most explored of the routine animal facility activities is the assessment of cage changes on behavioral and physiological outcomes. A cage change, although necessary, is effectively the manual removal of animals from an environment with familiar visual and olfactory cues to a clean cage. The novel environment combined with the handling has been shown to effect various parameters including sleep and activity patterns (Pernold et al., 2019) but also corticosterone and glucose levels (Balcombe, 2006; Ghosal et al., 2015). To understand if behavioral changes are apparent after the changing of housing, we compared activity and RDI of cages on the six subsequent days after a cage change compared to the day prior. With the length of our study, we were able to assess this up to 17 months of age. The analysis of light phase activity showed a consistent, significant elevation of activity on the day where a full cage change occurred. Although in different months, other days post cage change, also showed increased activity there was no consistent pattern and it was likely an outcome of the sheer amount of data analyzed. Dark phase activity presented no real trends that coincide with the cage changes, with what appears to be just differences in activity on different days in the week unrelated to cage change days. This is consistent with other published reports (Pernold et al., 2019; Golini et al., 2020). Additionally, our study reveals that mice do not habituate to the cage change, with significantly increased daytime activity post-cage change continuing up to 17 months of age. Only one study prior to ours has looked at the impact of RDI on cage change (Golini et al., 2020). In that study RDI on weekdays, cage change days and weekends were similar, after which they concluded that RDI is not impacted by cage changing or other procedures performed during daytime. Our study consistently shows over 17 months that RDI, like activity, is significantly increased on the day of cage change. This shows that animal activity is more irregular in the daytime of the cage change day compared to the previous day, when they are presumably resting as they have not been disturbed. Taken together these results indicate that cage changes should be avoided on the day of experimentation as the effect on activity and RDI can be a confounder to other investigations.

Automated home cage monitoring continues to aid our understanding of laboratory animal behavior, and we have shown here that it can be used to refine studies and diagnose the appearance of abnormal behaviors earlier. Our characterization of the DVC^®^ on such a long-term study has led us to conclude that from the standpoint of activity and RDI, further investigations should be performed to understand adequate acclimatization times after significant travel, as animals still display significantly altered diurnal patterns of behavior even 5 days after arrival. Aging of animals follow three distinct phases with altered profiles of activity and RDI largely reducing with age prior to an advanced rebound return of youthful activity levels. Despite these different phases with advanced age, habituation to cage change does not occur and animals continued to have heightened activity and irregular rest on the day of a cage change for the entire study period of 17 months. Therein it may be advisable to delay experimentation, especially for behavioral studies to later than the standard 5-day acclimatization and avoid experimentation days coinciding with cage change days. Crucially, the DVC^®^ was able to detect bouts of stereotypic behavior in group housed cages and should be used to monitor abnormal spikes of activity to allow early intervention. The use of HCM systems should be considered particularly for long term housing of animals, the assessment of behavioral phenotypes and refinement of laboratory animal care.

## Data Availability

The datasets presented in this article are not readily available because the raw data supporting the conclusions of this article is GSK internal data and therefore remains proprietary. Requests to access the datasets should be directed to Abdul-Azim Hassan, abdul-azim.x.hassan@gsk.com.
